# Supplementary material for: CircZXDC Promotes Vascular Smooth Muscle Cell Transdifferentiation via Regulating miRNA-125a-3p/ABCC6 in Moyamoya Disease
Source: Cells. 2022 Nov 26;11(23):3792. doi: 10.3390/cells11233792 (PMC9741004; doi:10.3390/cells11233792)
Supplement: Supplementary file 1 [file cells-11-03792-s001.zip › cells-1990060-supplementary.pdf]

Supplementary Figure S1

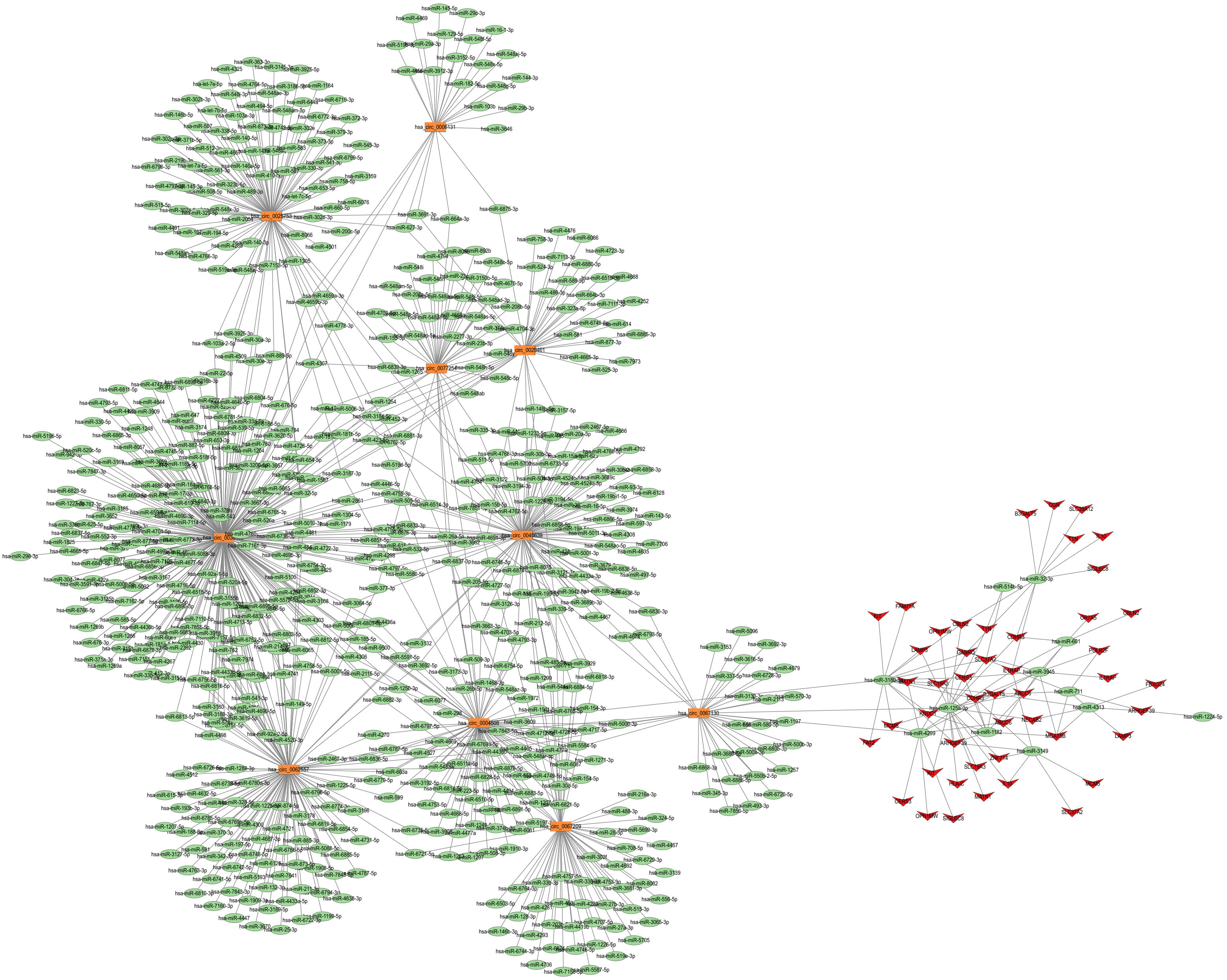

Supplementary Figure S1. The network of circRNAs/miRNAs/mRNAs in MMD based on RNA-seq datasets. The figure denotes the top 10 up-regulated circRNAs and potential interacting miRNAs, as well as the mRNAs, using circBase and Targetscan to establish the relationship.

Supplementary Figure S2

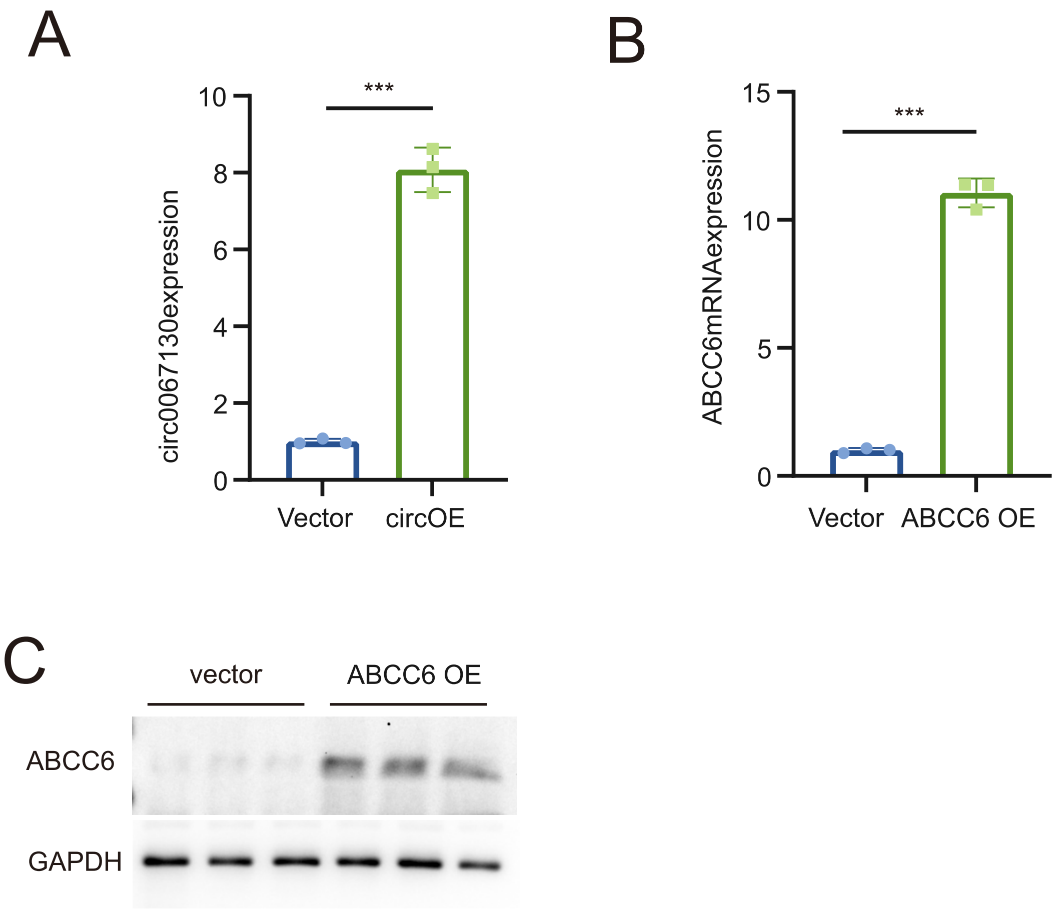

Supplementary Figure S2. Validation results of overexpression. (A) RT-qPCR results of VSMCs transfected with circZXDC overexpression plasmids compared to the vector. (B) RT-qPCR results of VSMCs transfected with ABCC6 overexpression plasmids compared to the vector. (C) Western blots of ABCC6 in VSMCs vector group and ABCC6 overexpression group.
